# Short Communication: Oral Administration of Heat-killed *Lactobacillus brevis* KB290 in Combination with Retinoic Acid Provides Protection against Influenza Virus Infection in Mice

**DOI:** 10.3390/nu12102925

**Published:** 2020-09-24

**Authors:** Shohei Satomi, Sofia Khanum, Poppy Miller, Shigenori Suzuki, Hiroyuki Suganuma, Axel Heiser, Sandeep K Gupta

**Affiliations:** 1 Department of Nature & Wellness Research, Innovation Division, KAGOME CO., LTD., 17 Nishitomiyama, Nasushiobara, Tochigi 329-2762, Japan; Shigenori_Suzuki@kagome.co.jp (S.S.); Hiroyuki_Suganuma@kagome.co.jp (H.S.); 2 AgResearch Ltd., Hopkirk Research Institute, Grasslands Research Centre, Private Bag 11008, Palmerston North 4442, New Zealand; Sofia.Khanum@agresearch.co.nz (S.K.); Poppy.Miller@agresearch.co.nz (P.M.); Axel.Heiser@agresearch.co.nz (A.H.)

**Keywords:** influenza virus, *Lactobacillus*, retinoic acid, combination effects

## Abstract

Influenza virus type A (IAV) is a seasonal acute respiratory disease virus with severe symptoms, and an effective preventive measure is required. Despite many reports describing the potentially protective effects of lactic acid bacteria, few studies have investigated the effects of nutritional supplement combinations. This study reports the effect of the combined intake of heat-killed *Lactobacillus brevis* KB290 (KB290) and vitamin A (VA) on mice challenged with a sublethal dose of IAV. For 2 weeks, five groups of mice were fed either placebo, KB290, VA, or a combination of KB290 and VA (KB290+VA). After subsequent IAV challenge, bodyweight and general health were monitored for up to 2 weeks. Viral titres were determined in the lungs of animal subgroups euthanised at days 3, 7, and 14 after IAV challenge. A significant loss was observed in the bodyweights of IAV-infected animals from day 1 post-IAV challenge, whereas the mice fed KB290+VA did not lose any weight after IAV infection, indicating successful protection from the infection. Additionally, mice in the KB290+VA group showed the highest reduction in lung viral titres. In conclusion, the combination of KB290 and VA could be a useful food supplement relevant for protection against seasonal influenza virus infection in humans.

## 1. Introduction

Seasonal influenza is a significant threat to human health that causes high levels of morbidity and mortality. Annual influenza epidemics are estimated to result in about 3 to 5 million cases of severe illness worldwide and about 290,000 to 650,000 respiratory deaths [1]. Clinical symptoms caused by influenza virus type A (IAV) infection often become severe in elderly individuals and infants as a result of a poor or weakened immune system [2,3]. Vaccination against IAV has been employed successfully, but this approach reduces the risk of medically attended influenza virus infection by only approximately 50%, and its effectiveness depends on the season, setting, and population subgroup [1]. Therefore, it is desirable to discover alternative strategies such as probiotics and food supplements that strengthen the innate and adaptive immune systems and promote protection to IAV infection.

Lactic acid bacteria (LAB) are historically important because of their utility in food preservation and fermentation; they can exert immunomodulatory effects in the respiratory tract and protect the host against IAV infection [4,5]. Previous studies have reported a reduction in IAV-induced weight loss in mice treated with LAB [4,6,7,8,9,10,11,12,13,14,15,16,17]. However, safety remains a major concern with the use of live microorganisms as probiotics because the potential risks include systemic infection, antibiotic resistance gene acquisition, and gut colonisation interference in neonates [18,19,20,21]. Recently, the use of heat-killed microorganisms as probiotics has attracted attention, and several studies have demonstrated the effects of such bacteria in modulating immune responses and enhancing the intestinal barrier function [22,23,24]. Moreover, it has been reported that both live and heat-killed lactobacillus strains show similar immune-modulatory properties [25,26,27,28], suggesting that heat-killed LAB could be used effectively without the safety concerns associated with the use of live LAB.

*Lactobacillus brevis* KB290 (KB290) is a plant-derived microorganism isolated from “*Suguki*”, a traditional pickle made in Kyoto, Japan. KB290 has been reported to be safe for human consumption, tolerant to gastrointestinal juices, and capable of improving human bowel function [29], as well as useful for early intervention in irritable bowel syndrome [30]. Furthermore, the KB290 ingestion has been shown to enhance interferon (IFN)-α production in humans [31]. It was previously reported that orally administered KB290 enhanced the cell-mediated cytotoxic activity of splenocytes, possibly via the activation of natural killer (NK) cells and/or CD8^+^ cytotoxic T cells [32], which alleviated clinical symptoms following influenza virus infection in mice [14], and reduced the incidence of influenza infection among schoolchildren [33].

Despite the large body of work describing the potentially protective effects of LAB alone, only a few studies have investigated the ability of nutritional supplement combinations to increase host protection against influenza. Vitamin A (VA) supplementation has been reported to modulate the intestinal cytokine response during viral infection and to contribute to both innate and adaptive immune responses [34,35,36,37]. Retinoic acid, a metabolite of VA, has been shown to promote mucosal immune responses by regulating gut cells [38,39,40]. In addition, retinoic acid has also been found to modulate the expression of retinoic acid-inducible gene-I (RIG-I), a viral pattern recognition receptor that is expressed during viral infection [41,42]. Furthermore, the oral administration of retinoic acid to mice fed a VA-deficient diet was reported to restore mucosal immunoglobulin A (IgA) responses to intranasal influenza virus vaccines [43].

Here, we provide evidence that oral coadministration of heat-killed KB290 and VA can induce protection against IAV infection in mice. To our knowledge, this is the first study to demonstrate the effects of a food nutrient combination on IAV infection.

## 2. Materials and Methods

### 2.1. Mice

Specific pathogen-free 7- to 8-week-old, female BALB/c mice were supplied by the AgResearch Small Animal Facility (Hamilton, New Zealand), and the study was conducted at AgResearch’s Ulyatt-Reid Facility (Palmerston North, New Zealand). Mice were housed in groups of 3–4 per cage in plastic cages on a 12-h light/dark cycle and fed mouse pellets

(Prolab^®^ RMH 1800; LabDiet, Richmond, IN, USA) and tap water (autoclaved under pressure at 121 °C for 15 min) ad libitum. The room temperature was kept at 21 °C, and the humidity was kept at 50%. All procedures involving the experimental use of animals were approved by the Grasslands Animal Ethics Committee, Palmerston North, New Zealand (approval number 14542).

### 2.2. Feeding Solution Preparation

KB290 was deposited as strain *L. brevis* JCM 17,312 in the Japan Collection of Microorganisms and has been maintained at the Research Institute, KAGOME CO., LTD. (Tochigi, Japan). Heat-killed and lyophilised powder of KB290 was used in the animal experiments. KB290 was suspended in phosphate-buffered saline (PBS) at a concentration of 5.41 × 10^10^ cells per mL immediately before use. Retinoic acid (Sigma, St. Louis, MO, USA) was suspended in food-grade canola oil (Pam’s Canola Oil, Made in Malaysia, purchased at Countdown, Palmerston North, New Zealand) at a concentration of 20 mg/mL.

### 2.3. Feeding Intervention

Mice were gavaged using 16ga polyurethane feeding tubes (Instech Laboratories, Plymouth Meeting, PA, USA) with 200 µL of feeding solution per mouse per day for 14 consecutive days prior to IAV challenge. Five groups containing 30 mice each received feeding solutions as follows: Normal group received placebo (185 µL of PBS + 15 µL of canola oil), the Control group also received placebo (185 µL of PBS + 15 µL of canola oil), VA group received 300 µg of retinoic acid (in 15 µL of canola oil + 185 µL of PBS), KB290 group received 10^10^ KB290 cells (in 185 µL of PBS + 15 µL of canola oil), and KB290+VA group received 10^10^ KB290 cells (in 185 µL of PBS) plus 300 µg of retinoic acid (in 15 µL of canola oil).

### 2.4. Influenza Virus

The challenge virus was a mouse-adapted strain of IAV, A/PR/8/34 (H1N1), prepared for a previous study by our group [14]. Briefly, the virus was propagated in the allantoic cavity of 10-day-old embryonated hen eggs. The allantoic fluid was harvested 3 days post-inoculation. The allantoic fluid supernatant was separated from the debris by centrifugation for 5 min at 500× *g* at 4 °C in a Heraeus Multifuge 3 S-R (ThermoFisher Scientific, Osterode am Harz, Germany); the resulting viral stocks were stored at −80 °C without the addition of preservatives. The 50% tissue culture infectious dose (TCID_50_) of the viral stock was determined to be 10^6.75^/mL by using the chicken red blood cell hemagglutination endpoint method in Madin–Darby canine kidney (MDCK) cells [44,45]. The LD_50_ (50% mouse lethal dose) for the intranasally inoculated virus was determined to be 2 × TCID_50_.

### 2.5. IAV Challenge

On the day after the continuous 14-day feeding period ended, all mice in four of the groups (Control, VA, KB290, and KB290+VA) were challenged with a sublethal dose of IAV. The Normal group was left unchallenged and housed in a separate room to prevent unintentional exposure to IAV. For the viral challenge, the mice were lightly anaesthetised by using a subcutaneous injection of 200 µL of ketamine/medetomidine containing 2.53 mg/mL ketamine (Ketamine injection; Parnell Laboratories NZ Ltd., Auckland, New Zealand; Product no. RVM A005925) and 0.03 mg/mL medetomidine hydrochloride (Domitor; Zoetis NZ, Auckland, New Zealand; Product no: RVM A06177). Anaesthetised mice were challenged intranasally with IAV by applying 10 µL of IAV in PBS per nostril (IAV concentration of 0.5× LD_50_ per 20 µL of PBS). After challenge, the mice were placed on a heat-pad (39 °C; Kent Scientific, Torrington, CT, USA) to maintain body temperature, and the anaesthesia was reversed by an injection of 100 µL of atipamezole hydrochloride (2.5 mg/mL Atipamezole; Zoetis NZ; Product no: RVM A006178).

### 2.6. Bodyweight, Body Temperature, and General Health Score

During the 14-day feeding period, mouse bodyweights were recorded every second day. After the IAV challenge, this measurement was recorded daily. Weight gain and loss were assessed using electronic scales (Sartorius, Göttingen, Germany). Animal welfare considerations mandated that mice who lost more than 20% of their bodyweight were to be euthanised by the method described below.

Body temperature was measured using a “No Touch + Forehead” thermometer (Braun, Kronberg im Taunus, Germany) using the method previously described and validated by Mei et al. [46]. The general health status (GHS) was assessed by using a five-point scale modified from Kawase et al. [47] to include signs of respiratory disease:

Score 5: The mouse is bright-eyed and alert, has a smooth coat with a sheen, responds to a stimulus, and shows interest in its environment. It has a normal breathing rate.

Score 4: Its fur is slightly ruffled with a loss of sheen in the coat, but the mouse remains alert and active. There is evidence of nasal irritation (intermittent nose rubbing/whisker twitch), but the mouse has a normal breathing rate.

Score 3: Its fur is noticeably ruffled, clumps have formed in part of the coat, and the mouse is less alert or active and less interested in its environment. The mouse is sneezing or coughing and continually rubbing its nose.

Score 2: The mouse is hunched over and sleepy, showing little interest in its environment, and its fur is clumped. The mouse is panting or has an audible “wet” breathing sound.

Score 1: The mouse is hunched over and sleepy, its fur has a “bottle brush” appearance (sticking out); the mouse is unreactive to environmental stimuli and its body and paws feel cold to the touch. The mouse has progressed from panting/hyperventilating to exhibiting a very slow respiration rate (gasping) and has an audible “wet” breathing sound.

### 2.7. Viral Titre

Of the 30 mice in each group, 10 mice were euthanised for post-mortem examination on each of the following post-IAV infection days: day 3, day 7, and day 14. Mice were euthanised by using CO_2_ inhalation and cervical dislocation. Euthanised mice were dissected, and their lungs were collected for viral titre analysis. The left lungs were transferred into 1.5-mL cryotubes, snap-frozen in liquid nitrogen, and transferred within 6 h to a freezer set at −80 °C for storage. The snap-frozen left lungs were later thawed and homogenised using a mixture of Zirconium beads, consisting of 0.5 g of 1-mm beads (Biospec Products, Bartlesville, OK, USA) and seven to eight 3-mm beads (Benchmark Scientific, Sayreville, NJ, USA). Homogenisation was performed in PBS supplemented with EDTA-free 1× protease inhibitor (Sigma, St. Louis, MO, USA), 100 U/mL penicillin, and 100 µg/mL streptomycin (Life Technologies, Auckland, New Zealand) using a TissueLyser II (Qiagen, Hilden, Germany) homogeniser set at 30 Hz for 5 min [48,49]. 

The titre of IAV in the supernatants from lung homogenates was determined by using the chicken red blood cell hemagglutination endpoint method in MDCK cells as described above. The value of the IAV titre was normalised against the total protein content of the sample and is expressed as the log_10_(TCID_50_). Total protein was quantified in clarified lung homogenates by using a BCA protein assay kit in accordance with the manufacturer’s instructions (ThermoFisher Scientific, Auckland, New Zealand).

### 2.8. Statistical Analysis

#### 2.8.1. Bodyweight Analysis

A generalized additive mixed-effects model (gam model from R package mgcv) [50] was fitted to the observed weights with the following covariates: fixed effects for the initial weight (weight when challenged with IAV) and treatment group; a spline for the time effect for each treatment; and random intercepts for each mouse ID and cage. The spline allowed the time effect to be a flexible shape, driven by the data. Estimated marginal mean (emm) curves were calculated using the R package emmeans [51]. Estimated marginal means were calculated by creating a grid of predicted weights for all animals at all times (using the mean initial weight) and averaging the resulting predictions within group and time. This processadjusted for the data imbalance caused by killing animals on days 3 and 7 for post-mortems (as it predicted what their weights would have been had they not been killed) and for the differing mean initial weights for animals in each group and time. Therefore, the emms provided unbiased comparisons between treatments. In general, raw means and emms are identical only when the data are fully balanced and no continuous covariates (e.g., initial weight) are used. Pairwise comparisons between treatments were reported for each sampled time and the resulting *p*-values were adjusted using Benjamini and Hochberg’s (BH) *p*-value adjustment [52]. 

#### 2.8.2. Viral Titre

The normalised log_10_(TCID_50_) viral titres were compared using pairwise permutation t-tests [53,54]. The data were not normally distributed, therefore a permutation t-test (pairwise.perm.t.test) was used from the RVAideMemoire R [55]. The permutation t-test compares the observed difference in mean for the two groups to an empirical null distribution. 

BH *p*-value adjustment [52] was applied to the *p*-values from all pairwise comparisons; statements of significant differences are based on *p*-values of <0.05 and are presented as the mean ± SEM unless indicated otherwise. Treatment group data were compared with the Normal and Control group data on day 3.

A total of three mice needed to be euthanised during the study because of animal welfare considerations. One mouse from the day 7 PM subgroup of the Control group was euthanised because of a non-study-related injury at day 1 post-IAV challenge, so this subgroup had nine mice for all measurements, and this was considered as a randomly missing data point for statistical analyses. Two mice were euthanised at day 11 post-IAV challenge from the day 14 PM subgroup of the Control group because these mice lost more than 20% of their bodyweight. This was likely a result of the IAV challenge. However, the lungs were retrieved from both animals, so the viral titres in this group were still measured from 10 animals as planned. The bodyweights for these two mice were still included in the bodyweight statistical analyses and were treated as day-14 samples.

## 3. Results

### 3.1. Bodyweight, General Health Score, and Body Temperature Changes

To examine the effect of orally administered KB290 and VA in combination or alone, the bodyweight, general health score (GHS), and body temperature of the animals were recorded for 2 weeks before and after IAV challenge.

**Figure 1:**
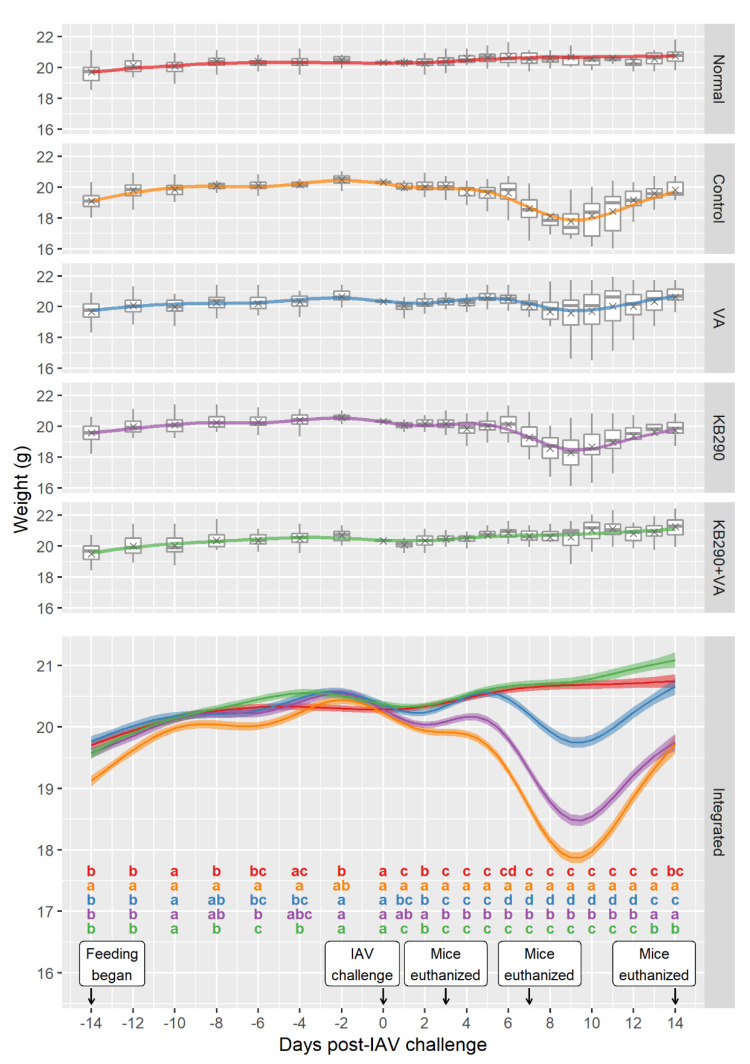
Bodyweights of mice with each treatment before and after Influenza virus type A (IAV) challenge. The estimated marginal mean curves (coloured by treatment) show bodyweight changes over time for a mouse of the average initial weight. The observed data were adjusted for initial weight by subtracting their individual initial weight and adding on the observed mean initial weight. The adjusted weights were plotted as grey boxplots with the mean adjusted weight at each time shown using a grey cross. Pairwise comparisons between the treatment groups were conducted at each observation time. The treatments are significantly different if they do not share a letter (a, b, c, and d) within each time (significance cut-off was 0.05 after using the BH *p*-value adjustment). The uncertainty ribbon on the combined plot shows the mean ± SE (standard error). Each group initially included 30 mice, 10 of which were euthanised after weighing on each of the following days: 3, 7, and 14 post-IAV challenge.

Significant differences in mouse bodyweights among groups were observed from day 1 for several treatments and day 7 for all treatments except Normal and KB290+VA after IAV challenge (Figure 1). The animals in the IAV-infected Control group had significant bodyweight loss from day 1 to 14 after IAV challenge compared with those in the non-infected Normal group, suggesting the establishment of IAV infection in these animals. The bodyweight loss in the KB290+VA group animals was significantly lower than that in the Control group, from all days after IAV challenge. In addition, no differences in mice bodyweight were observed between the KB290+VA group and the Normal group for any period post IAV challenge except for day 13, indicating that KB290+VA-fed mice maintained their bodyweight after IAV infection similarly to the uninfected mice. Furthermore, the bodyweight loss in the VA and KB290 groups were also significantly higher to the Normal group between day 7 and day 12 and between day 1 and day 14, respectively. The bodyweight loss of the VA group was significantly lower compared with that of the Control group for all days but was significantly higher compared with that of the KB290+VA group between day 6 and day 14 post-IAV-challenge. The bodyweight loss of the KB290 group was significantly lower compared with that of the Control group between day 3 and day 12 but was significantly higher compared with that of the KB290+VA group for all days after IAV challenge.

Some non-significant differences were observed in the GHS of animals in the Control and KB290 groups after IAV challenge. The GHS values for the animals in the Control group trended lower than those for the KB290 group mice (data not shown). No differences in the GHS after IAV challenge were observed between any other groups. In addition, no differences were observed in the body temperatures of the animals among any of the treatment groups (data not shown).

Overall, the result of bodyweight change suggests that the synergistic effect of combined feeding of KB290 and VA to mice before influenza infection resulted in better health status compared with mice fed KB290 or VA alone.

### 3.2. Viral Titre

**Figure 2:**
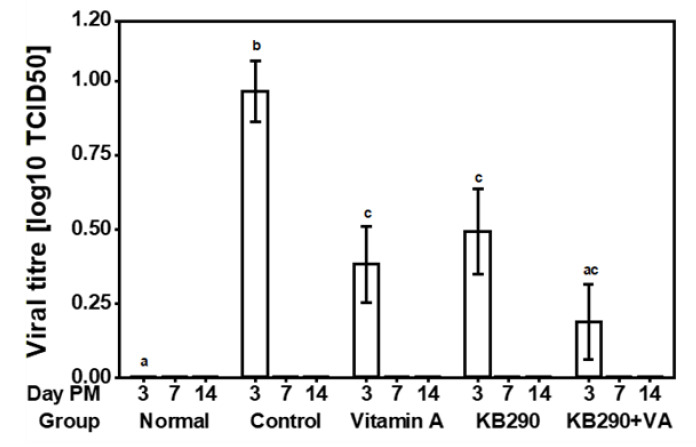
IAV virus titres in murine lungs. Viral titres in the lungs, presented as the TCID_50_ (50% tissue culture infectious dose). This measurement is shown for the five treatment groups at days 3, 7, and 14 post-IAV challenge (*n* = 10). Different letters (a, b, and c) indicate significant differences (*p* < 0.05, using the BH p-value adjustment) between the groups on day 3.

On day 3 post-IAV challenge, the lung virus titres in the non-infected Normal and IAV-infected Control groups were 0 ± 0 log_10_ TCID_50_ (mean ± SEM) and 0.966 ± 0.321 log_10_ TCID_50_ (mean ± SEM), respectively; and were found to be significantly different (*p* = 0.002) (Figure 2).

Significantly lower viral titres were observed in lungs of the VA (*p* = 0.01) and KB290 (*p* = 0.04) fed mice compared to the Control group, but these viral tires were significantly higher for the VA (*p* = 0.05) and KB290 (*p* = 0.03) groups compared to the Normal group on day 3. Mice in the KB290+VA group showed the highest reduction in lung viral titre compared with the Control group (*p* = 0.01), but no significant difference was observed in viral titre compared to the Normal group (*p* = 0.47). No virus was detected in the lungs of the mice in any of the groups at days 7 and 14 post-IAV challenge.

## 4. Discussion

Here, we found that the ingestion of a combination of heat-killed KB290 and VA suppressed bodyweight loss and reduced the lung viral titre in mice challenged with a sublethal dose of IAV. Challenging mice with lethal doses of IAV results in markedly different immunopathology compared with challenging them with sublethal doses [56,57,58,59,60]. Sublethal infection with IAV is physiologically relevant because it can closely mimic seasonal influenza infection in humans. In this study, the viral loads can be detected only after 72 h in mice infected with a sublethal dose of IAV were consistent with previous findings, indicating that mild IAV infection was established in these animals [60,61]. We demonstrated that, after infection with a sublethal dose of IAV, mice pre-treated with a combination of heat-killed KB290 and VA had a significantly lower viral load. A reduction in influenza virus in the lungs has been reported as a result of consuming other Lactobacilli, namely *L. acidophilus* [62], *L. casei* [63], *L. delbrueckii* [64], *L. gasseri* [6,13], *L. rhamnosus* [65], and *L. plantarum* [4,10].

Infection with a lethal dose of influenza virus results in an overwhelming release of proinflammatory cytokines and severe bodyweight loss of up to 35%, causing high mortality. Sublethal influenza virus doses have resulted in bodyweight losses of up to 20% and lower mortality compared with lethal doses [56,59]. To simulate naturally occurring influenza virus infections in humans, this study investigated mice challenged with a sublethal dose of IAV. The observed bodyweight losses in the IAV-infected and untreated groups were comparable with those of a previously published report [56]. Feeding of VA by mice prior to IAV challenge led to a marked reduction in bodyweight loss, and feeding of KB290+VA completely prevented IAV infection-induced bodyweight loss. A reduction in bodyweight loss after treating mice with various LAB species, such as live *L. brevis* KB290 [14], *L. bulgaricus* [17], *L. gasseri* [6,13], *L. paracasei* [15], *L. pentosus* [8], *L. plantarum* [4,7,10,11,12,16], and *L. rhamnosus* [9], has been reported previously. A complete maintenance of bodyweight despite IAV infection, as observed in this study, has so far been reported only once after an intranasal pre-treatment of mice with heat-killed *L. casei* [63]. Focusing on the KB290 strain, the present study showed that heat-killed KB290 resulted in suppressing the bodyweight loss in IAV-challenged mice. This finding was in agreement with a previous report, which demonstrated that live KB290 can reduce the influence of IAV in mice [14]. In addition, both live and heat-killed KB290 have been shown to enhance cell-mediated cytotoxic activity in mice [66]. Taken together, these findings suggest that both live and heat-killed forms of KB290, could be effective in providing protection against IAV infection by possibly enhancing the function of immune cells.

The mechanisms by which feeding of a combination of heat-killed KB290 and VA suppressed IAV infection are not clear. Our previous report showed that live KB290 induced IFN-α and IgA in the mice challenged with IAV [14]. In addition, retinoic acid has been reported to be related to RIG-I-mediated production of IFN-α [41,42] and IgA [43]. Compared with the intake of VA or KB290 alone, their combined administration strongly suppressed influenza infection, possibly by improving the local immune responses in the lungs. Future work should focus on understanding the enhancing effects of this combination on innate and adaptive immune pathways both locally and systemically. 

Although retinoic acid contributes to various immune processes [67], excessive intake of VA can be toxic [68]; this creates an obstacle for the human application of VA consumption as a potential influenza preventative. The Non-Observed Adverse Effect Level and Lowest Observed Adverse Effect Level of VA have been reported as 6000 and 13,500 μg/day, respectively [69]. Based on the current study, it is estimated that 3690 μg/day of VA equivalent dose in adult humans will be required [70]. While this amount is slightly higher than the upper recommended limit of VA for daily intake according to the National Institute of Health [71], a much lower dose of VA in combination with KB290 could be sufficient in the gut to show protection against seasonal influenza.

As a safer plan for humans, β-carotene is the most appropriate candidate for VA sources because it is a pro-VA present in plant-derived foods such as carrots and its efficiency for VA production in humans is thought to be regulated by an individual’s VA status [72]. No serious side effects of β-carotene have been reported, except when a large amount of it was ingested as a supplement under high oxidative stress conditions, like smoking [73]. Therefore, it is considered as an ideal alternative to retinoic acid for human consumption. Further studies will be also required to establish the effective dose of VA in the form of β-carotene in combination with KB290 in humans.

In summary, the results presented here provide evidence for the efficacy of oral administration of the combination of heat-killed *Lactobacillus brevis* KB290 and VA for protecting mice against a sublethal dose of IAV. These findings suggest that the combination of KB290 and VA could be a useful food supplement to provide protection against seasonal influenza virus infections in humans.
